# Supplementary material for: The Systems Biology Research Tool: evolvable open-source software
Source: BMC Syst Biol. 2008 Jun 29;2:55. doi: 10.1186/1752-0509-2-55 (PMC2446383; doi:10.1186/1752-0509-2-55)
Supplement: Additional file 1 — SBRT Archive. An archive of the current version of the Systems Biology Research Tool. [file 1752-0509-2-55-S1.zip › sbrt-1.4.0/doc/developers_guide/api/overview-tree.html]

Class Hierarchy


|  |  |  |
| --- | --- | --- |
| **Overview** \| Package \| Class \| Use \| **Tree** \| **Deprecated** \| **Index** \| **Help** | |  |
| PREV   NEXT | **FRAMES**    **NO FRAMES**     **All Classes** |  |


---


## Hierarchy For All Packages

**Package Hierarchies:**: sbrt, sbrt.ext, sbrt.ext.cplex, sbrt.ext.glpk, sbrt.ext.mma, sbrt.ext.mma.algebra, sbrt.ext.R, sbrt.ext.R.stat, sbrt.kernel, sbrt.kernel.fba, sbrt.kernel.fba.analysis, sbrt.kernel.fba.comp, sbrt.kernel.fba.proc, sbrt.kernel.io, sbrt.kernel.math, sbrt.kernel.math.algebra, sbrt.kernel.math.algebra.proc, sbrt.kernel.math.comb.comp, sbrt.kernel.math.comb.proc, sbrt.kernel.math.geom, sbrt.kernel.math.geom.comp, sbrt.kernel.math.geom.proc, sbrt.kernel.math.graph\_theory, sbrt.kernel.math.graph\_theory.comp, sbrt.kernel.math.graph\_theory.proc, sbrt.kernel.math.optimization, sbrt.kernel.math.stat, sbrt.kernel.math.stat.comp, sbrt.kernel.math.stat.proc, sbrt.kernel.util, sbrt.kernel.utilities.proc, sbrt.shell, sbrt.shell.gui, sbrt.shell.io, sbrt.shell.io.fba, sbrt.shell.mng, sbrt.shell.mng.algebra, sbrt.shell.mng.comb, sbrt.shell.mng.fba, sbrt.shell.mng.geom, sbrt.shell.mng.graph\_theory, sbrt.shell.mng.stat, sbrt.shell.mng.utilities, sbrt.shell.proc.fba, sbrt.shell.proc.graph\_theory, sbrt.shell.text, sbrt.shell.text.chem, sbrt.shell.text.fba, sbrt.shell.text.math

---

## Class Hierarchy

- java.lang.Object
  - sbrt.kernel.fba.comp.**AbstractCvComputer** (implements sbrt.kernel.fba.comp.FluxCvComputer)
    - sbrt.kernel.fba.comp.**AbstractFluxIntervalComputer** (implements sbrt.kernel.fba.comp.FluxIntervalComputer)
      - sbrt.kernel.fba.comp.**FastMsFluxRangeComputer**- sbrt.kernel.fba.comp.**MsFluxIntervalComputer**- sbrt.kernel.fba.comp.**ReverseRxnFluxIntervalComputer**- sbrt.kernel.fba.comp.**SimpleFluxIntervalComputer**
      - sbrt.kernel.fba.comp.**CdHarFluxVectorComputer**- sbrt.kernel.fba.comp.**RxnDeletionComputer**
    - sbrt.kernel.math.**AbstractLinearComb.Term**<V> (implements sbrt.kernel.math.LinearComb.Term<V>)
      - sbrt.kernel.math.**HashLinearComb.Term**<V>
      - sbrt.shell.text.**AbstractLinkedHashSetFormat**<E> (implements sbrt.shell.text.LinkedHashSetFormat<E>)
        - sbrt.shell.text.**AmpersandSetFormat**<E>- sbrt.shell.text.**PipeSetFormat**<E>- sbrt.shell.text.**TabSetFormat**<E>- sbrt.shell.text.**WhitespaceSetFormat**<E>
        - sbrt.shell.text.**AbstractListFormat**<E> (implements sbrt.shell.text.ListFormat<E>)
          - sbrt.shell.text.**PipeListFormat**<E>- sbrt.shell.text.**TabListFormat**<E>- sbrt.shell.text.**WhitespaceListFormat**<E>
          - sbrt.shell.text.**AbstractMapEntryFormat**<K,V> (implements sbrt.shell.text.MapEntryFormat<K,V>)
            - sbrt.shell.text.**ColonMapEntryFormatV1**<K,V>- sbrt.shell.text.**EqualsMapEntryFormatV1**<K,V>
            - sbrt.shell.text.**AbstractMapFormat**<K,V> (implements sbrt.shell.text.MapFormat<K,V>)
              - sbrt.shell.text.**AppInputFileLineFormatVersion1** (implements sbrt.shell.text.AppInputFileLineFormat)- sbrt.shell.text.**ColonMapFormatV1**<K,V>- sbrt.shell.text.fba.**ConstraintsFileLineFormatV1** (implements sbrt.shell.text.fba.ConstraintsFileLineFormat<E>)- sbrt.shell.text.fba.**ConstraintsFileLineFormatV2** (implements sbrt.shell.text.fba.ConstraintsFileLineFormat<E>)- sbrt.shell.text.**EqualsMapFormatV1**<K,V>- sbrt.shell.text.fba.**FluxCapFormatV1** (implements sbrt.shell.text.fba.FluxCapFormat)- sbrt.shell.text.fba.**FluxomeSolFileLineFormatV1** (implements sbrt.shell.text.fba.FluxomeSolutionFileLineFormat)- sbrt.shell.text.fba.**FluxVectorFormatV1** (implements sbrt.shell.text.fba.FluxVectorFormat)- sbrt.shell.text.fba.**IrfFormatV1**
              - sbrt.shell.io.**AbstractMapInputFile**<K,V> (implements sbrt.shell.io.MapTextInputFile<K,V>)
                - sbrt.shell.io.**ComiFile**<K,V>- sbrt.shell.io.**RomiFile**<K,V>
                    - sbrt.shell.io.**ProcessInputFile** (implements sbrt.shell.mng.ManagerInput<K,V>)
                - sbrt.shell.io.**AbstractMapOutputFile**<K,V> (implements sbrt.shell.io.MapTextOutputFile<K,V>)
                  - sbrt.shell.io.**ComoFile**<K,V>- sbrt.shell.io.**RomoFile**<K,V>
                  - sbrt.kernel.math.algebra.**AbstractMdMatrix**<R,C> (implements sbrt.kernel.math.algebra.DoubleMatrixBuilder<R,C>)
                    - sbrt.kernel.math.algebra.**AbstractSmdMatrix**<R,C> (implements sbrt.kernel.math.algebra.SmdMatrix<R,C>)
                      - sbrt.kernel.math.algebra.**HashMatrix**<R,C>- sbrt.kernel.math.algebra.**RowOrTreeMatrix**<R,C>- sbrt.kernel.math.algebra.**TreeMatrix**<R,C>
                    - sbrt.kernel.math.**AbstractPolynomial**<V> (implements sbrt.kernel.math.Polynomial<V>)
                      - sbrt.kernel.math.**AbstractLinearComb**<V> (implements sbrt.kernel.math.LinearComb<V>)
                        - sbrt.kernel.math.**HashLinearComb**<V>
                      - sbrt.shell.mng.**AbstractProcessManager** (implements sbrt.shell.mng.ProcessManager<K,V>)
                        - sbrt.shell.mng.fba.**BiggSbmlReaderManager** (implements sbrt.shell.mng.ShellProcessManager<K,V>)- sbrt.shell.mng.fba.**BiggSbmlTranslaterManager** (implements sbrt.shell.mng.ShellProcessManager<K,V>)- sbrt.shell.mng.geom.**CdHarManager** (implements sbrt.shell.mng.KernelProcessManager<K,V>)- sbrt.shell.mng.stat.**CorrelationManager** (implements sbrt.shell.mng.KernelProcessManager<K,V>)- sbrt.shell.mng.graph\_theory.**CycleIdManager** (implements sbrt.shell.mng.KernelProcessManager<K,V>)- sbrt.shell.mng.fba.**FbaManager** (implements sbrt.shell.mng.KernelProcessManager<K,V>)
                                    - sbrt.shell.mng.fba.**CatalystDeletionManager**- sbrt.shell.mng.fba.**CvCatalystDeletionManager**- sbrt.shell.mng.fba.**CvFbaOptManager**- sbrt.shell.mng.fba.**CvMsFluxIntervalManager**- sbrt.shell.mng.fba.**CvObjectiveFunctionManager**- sbrt.shell.mng.fba.**CvRevRxnFluxIntervalManager**- sbrt.shell.mng.fba.**CvRxnDeletionManager**- sbrt.shell.mng.fba.**CvSimpleFluxIntervalManager**- sbrt.shell.mng.fba.**ExtremeCurrentManager**- sbrt.shell.mng.fba.**FbaOptManager**- sbrt.shell.mng.fba.**FbaSolverManager**- sbrt.shell.mng.fba.**FluxActivityManager**- sbrt.shell.mng.fba.**FluxCapManager**- sbrt.shell.mng.fba.**FluxPlasticityManager**- sbrt.shell.mng.fba.**InitialPointManager**- sbrt.shell.mng.fba.**IntervalVariationManager**- sbrt.shell.mng.fba.**MsFluxIntervalManager**- sbrt.shell.mng.fba.**MsFluxomeReducerManager**- sbrt.shell.mng.fba.**NetworkInfoManager**- sbrt.shell.mng.fba.**ObjectiveFunctionManager**- sbrt.shell.mng.fba.**RandomConstraintsManager**- sbrt.shell.mng.fba.**RandomEnvManager**- sbrt.shell.mng.fba.**RandomFluxVectorManager**
                                                                                  - sbrt.shell.mng.fba.**CdHarFluxVectorManager**
                                                                                  - sbrt.shell.mng.fba.**RandomObjectiveManager**- sbrt.shell.mng.fba.**ReverseRxnFluxIntervalManager**- sbrt.shell.mng.fba.**RxnDeletionManager**- sbrt.shell.mng.fba.**SimpleFluxIntervalManager**- sbrt.shell.mng.fba.**SlpCycleIdManager**- sbrt.shell.mng.fba.**StoichVariationManager**- sbrt.shell.mng.fba.**SvCdHarFluxVectorManager**- sbrt.shell.mng.fba.**SvInitialPointManager**- sbrt.shell.mng.fba.**WwFluxomeReducerManager**
                                    - sbrt.shell.mng.utilities.**IntervalComparisonManager** (implements sbrt.shell.mng.KernelProcessManager<K,V>)- sbrt.shell.mng.stat.**KendallTauManager** (implements sbrt.shell.mng.KernelProcessManager<K,V>)- sbrt.shell.mng.algebra.**LinearCombConverterManager** (implements sbrt.shell.mng.ShellProcessManager<K,V>)- sbrt.shell.mng.algebra.**LinearSolverManager** (implements sbrt.shell.mng.KernelProcessManager<K,V>)- sbrt.shell.mng.stat.**MannWhitneyUManager** (implements sbrt.shell.mng.KernelProcessManager<K,V>)- sbrt.shell.mng.algebra.**MatrixConverterManager** (implements sbrt.shell.mng.ShellProcessManager<K,V>)- sbrt.shell.mng.fba.**MetatoolFileWriterManager** (implements sbrt.shell.mng.ShellProcessManager<K,V>)- sbrt.shell.mng.fba.**MmaNotebookManager**- sbrt.shell.mng.algebra.**MultipleVectorsConverterManager** (implements sbrt.shell.mng.ShellProcessManager<K,V>)- sbrt.shell.mng.fba.**PalssonSbmlReaderManager** (implements sbrt.shell.mng.ShellProcessManager<K,V>)- sbrt.shell.mng.fba.**PalssonSbmlTranslaterManager** (implements sbrt.shell.mng.ShellProcessManager<K,V>)- sbrt.shell.mng.graph\_theory.**PathIdManager** (implements sbrt.shell.mng.KernelProcessManager<K,V>)- sbrt.shell.mng.fba.**RxnRemovalManager**- sbrt.shell.mng.fba.**SbmlWriterManager** (implements sbrt.shell.mng.ShellProcessManager<K,V>)- sbrt.shell.mng.fba.**SimpleRxnFileTransManager** (implements sbrt.shell.mng.ShellProcessManager<K,V>)- sbrt.shell.mng.comb.**SingleElementUnionManager** (implements sbrt.shell.mng.KernelProcessManager<K,V>)- sbrt.shell.mng.algebra.**SingleVectorConverterManager** (implements sbrt.shell.mng.ShellProcessManager<K,V>)- sbrt.shell.mng.comb.**StrictSingleElementUnionManager** (implements sbrt.shell.mng.KernelProcessManager<K,V>)- sbrt.shell.mng.graph\_theory.**UniqueCycleIdManager** (implements sbrt.shell.mng.ShellProcessManager<K,V>)- sbrt.shell.mng.utilities.**VariableParticipationManager** (implements sbrt.shell.mng.KernelProcessManager<K,V>)- sbrt.shell.mng.utilities.**VectorComparisonManager** (implements sbrt.shell.mng.KernelProcessManager<K,V>)
                        - sbrt.kernel.util.**AbstractProgressMonitor** (implements sbrt.kernel.util.MutableProgressMonitor)
                          - sbrt.kernel.util.**DiscreteProgressMonitor**- sbrt.kernel.util.**PercentProgressMonitor**
                          - sbrt.kernel.io.**AbstractResultsManager**<R> (implements sbrt.kernel.io.ResultsManager<T>)
                            - sbrt.shell.mng.fba.**ExtremeCurrentManager.Rm**- sbrt.kernel.io.**ListResultsManager**<R> (implements java.lang.Iterable<T>)- sbrt.kernel.io.**SingleRm**<R>
                            - sbrt.kernel.fba.**AbstractRxn** (implements sbrt.kernel.fba.ChemRxn)
                              - sbrt.kernel.fba.**IrrevRxn**- sbrt.kernel.fba.**ReversibleRxn**
                              - sbrt.shell.io.**AbstractSbrtFile**- sbrt.shell.text.fba.**BiggBracketParserV1**- sbrt.shell.text.fba.**BiggRxnNodeParser**- sbrt.shell.proc.fba.**BiggSbmlReader**- sbrt.shell.proc.fba.**BiggSbmlTranslater**- sbrt.kernel.util.**BooleanRelation**<E> (implements java.lang.Iterable<T>)
                                          - sbrt.kernel.util.**And**<E>- sbrt.kernel.util.**Or**<E>
                                          - sbrt.shell.text.**BoolRelFormatV1**<E> (implements sbrt.shell.text.SimpleFormat<T>)- sbrt.shell.text.math.**BoundedIntervalFormatV1** (implements sbrt.shell.text.math.IntervalFormat)- sbrt.shell.text.chem.**CatalystFormatV1** (implements sbrt.shell.text.chem.CatalystFormat)- sbrt.shell.text.chem.**CatalystFormatV2** (implements sbrt.shell.text.chem.CatalystFormat)- sbrt.shell.text.fba.**CatalystListFormatV1** (implements sbrt.shell.text.fba.CatalystListFormat)- sbrt.shell.text.fba.**CatalystSetFormatV1** (implements sbrt.shell.text.fba.CatalystSetFormat)- sbrt.shell.text.fba.**CatalystVerifier** (implements sbrt.shell.text.chem.CatalystFormat)- sbrt.kernel.math.geom.comp.**CdHarComputer**<V>- sbrt.kernel.math.geom.comp.**CdHarComputer.Results**- sbrt.kernel.fba.comp.**CdHarFluxVectorComputer.Results**- sbrt.kernel.math.geom.proc.**CdHarProcess**<V> (implements sbrt.kernel.MonitoredProcess<R>)- sbrt.shell.text.chem.**ChemSpeciesFormatV1** (implements sbrt.shell.text.chem.ChemSpeciesFormat)- sbrt.shell.text.fba.**ChemSpeciesVerifier** (implements sbrt.shell.text.chem.ChemSpeciesFormat)- sbrt.shell.text.math.**CoeffFormatV1** (implements sbrt.shell.text.math.CoeffFormat)- sbrt.shell.text.math.**CoeffFormatV2** (implements sbrt.shell.text.math.CoeffFormat)- sbrt.shell.io.fba.**CofoofSource** (implements java.util.Iterator<E>)- sbrt.shell.io.fba.**ColumnOrFbaOptOutputFile** (implements sbrt.shell.io.fba.FbaOptOutputFile)- sbrt.shell.io.**ComiFileSource**<K,V> (implements java.util.Iterator<E>)- sbrt.shell.io.**ComoFileSource**<K,V> (implements java.util.Iterator<E>)- java.awt.Component (implements java.awt.image.ImageObserver, java.awt.MenuContainer, java.io.Serializable)
                                                                                  - java.awt.Container
                                                                                    - javax.swing.JComponent (implements java.io.Serializable)
                                                                                      - javax.swing.JPanel (implements javax.accessibility.Accessible)
                                                                                        - sbrt.shell.gui.**SbrtGui** (implements java.awt.event.ActionListener)- sbrt.shell.gui.**TextPaneGui** (implements java.awt.event.ActionListener)
                                                                                  - sbrt.kernel.math.stat.proc.**CorrelationAnalysis**<V,T> (implements sbrt.kernel.KernelProcess<T>)- sbrt.shell.text.math.**CorrResFormatterV1** (implements sbrt.shell.text.math.CorrResultsFormatter<T>)- sbrt.ext.cplex.**CplexSolver**<R,C> (implements sbrt.kernel.math.optimization.LinearProgramSolver<R,C>, sbrt.kernel.math.optimization.QuadraticProgramSolver<R,C>)- sbrt.kernel.math.graph\_theory.proc.**CycleIdProcess**<N> (implements sbrt.kernel.MonitoredProcess<R>)- sbrt.kernel.math.graph\_theory.**DirectedGraph**<N>- sbrt.kernel.math.graph\_theory.**DirectedGraph.Edge**<N>- sbrt.shell.text.math.**DirEdgeFormatV1**<N> (implements sbrt.shell.text.math.DirEdgeFormat<N>)- sbrt.kernel.math.**DoubleEquiv** (implements sbrt.kernel.math.EquivTester<T>)- sbrt.shell.text.**DoubleFormatV1** (implements sbrt.shell.text.DoubleFormat)- sbrt.shell.text.**DoubleFormatV2** (implements sbrt.shell.text.DoubleFormat)- sbrt.ext.R.**DummyConsole** (implements org.rosuda.JRI.RMainLoopCallbacks)- sbrt.shell.io.**EndOfFile** (implements java.io.Serializable)- sbrt.kernel.math.geom.comp.**EuclideanDist**- sbrt.kernel.fba.**ExchangeRxn** (implements sbrt.kernel.fba.ChemRxn)
                                                                                                              - sbrt.kernel.fba.**SinkRxn**- sbrt.kernel.fba.**SourceRxn**
                                                                                                              - sbrt.kernel.fba.comp.**FbaOptComputer** (implements sbrt.kernel.fba.comp.FluxCvComputer)- sbrt.kernel.fba.comp.**FbaOptComputer.Results**- sbrt.shell.text.fba.**FbaOptFileLineFormatV1** (implements sbrt.shell.text.fba.FbaOptFileLineFormat)- sbrt.shell.text.fba.**FbaOptHeaderFormatterV1** (implements sbrt.shell.text.fba.FbaOptHeaderFormatter)- sbrt.shell.text.fba.**FbaOptHeaderParserV1** (implements sbrt.shell.text.fba.FbaOptHeaderParser)- sbrt.kernel.fba.proc.**FbaProcess**<R> (implements sbrt.kernel.KernelProcess<T>)
                                                                                                                          - sbrt.kernel.fba.proc.**CatalystDeletionProcess** (implements sbrt.kernel.MonitoredProcess<R>)- sbrt.kernel.fba.proc.**CdHarFluxVectorProcess** (implements sbrt.kernel.MonitoredProcess<R>)- sbrt.kernel.fba.proc.**CvCatalystDeletionProcess** (implements sbrt.kernel.MonitoredProcess<R>)- sbrt.kernel.fba.proc.**CvFbaOptimizationExperiment** (implements sbrt.kernel.MonitoredProcess<R>)- sbrt.kernel.fba.proc.**CvFbaOptProcess**<R>
                                                                                                                                    - sbrt.kernel.fba.proc.**CvFluxIntervalProcess** (implements sbrt.kernel.MonitoredProcess<R>)- sbrt.kernel.fba.proc.**SvCdHarFluxVectorProcess** (implements sbrt.kernel.MonitoredProcess<R>)- sbrt.kernel.fba.proc.**SvInitialPointProcess** (implements sbrt.kernel.MonitoredProcess<R>)
                                                                                                                                    - sbrt.kernel.fba.proc.**CvObjectiveFunctionProcess** (implements sbrt.kernel.MonitoredProcess<R>)- sbrt.kernel.fba.proc.**CvRxnDeletionProcess** (implements sbrt.kernel.MonitoredProcess<R>)- sbrt.kernel.fba.proc.**CycleIdProcess** (implements sbrt.kernel.MonitoredProcess<R>)- sbrt.kernel.fba.proc.**ExtremeCurrentProcess** (implements sbrt.kernel.MonitoredProcess<R>)- sbrt.kernel.fba.proc.**FbaOptimizationProcess**- sbrt.kernel.fba.proc.**FbaSolverProcess**- sbrt.kernel.fba.proc.**FluxActivityProcess** (implements sbrt.kernel.MonitoredProcess<R>)- sbrt.kernel.fba.proc.**FluxCapProcess** (implements sbrt.kernel.MonitoredProcess<R>)- sbrt.kernel.fba.proc.**FluxIntervalProcess** (implements sbrt.kernel.MonitoredProcess<R>)- sbrt.kernel.fba.proc.**FluxomeReducerProcess**<T> (implements sbrt.kernel.MonitoredProcess<R>)- sbrt.kernel.fba.proc.**FluxPlasticityProcess** (implements sbrt.kernel.MonitoredProcess<R>)- sbrt.kernel.fba.proc.**InitialPointGenerator**- sbrt.kernel.fba.proc.**NetworkInfoProcess**- sbrt.kernel.fba.proc.**ObjectiveFunctionAnalysis** (implements sbrt.kernel.MonitoredProcess<R>)- sbrt.kernel.fba.proc.**RandomConstraintsGeneratorV1** (implements sbrt.kernel.MonitoredProcess<R>)- sbrt.kernel.fba.proc.**RandomEnvGeneratorV1** (implements sbrt.kernel.MonitoredProcess<R>)- sbrt.kernel.fba.proc.**RandomObjectiveGenerator** (implements sbrt.kernel.MonitoredProcess<R>)- sbrt.kernel.fba.proc.**RxnDeletionProcess** (implements sbrt.kernel.MonitoredProcess<R>)- sbrt.kernel.fba.proc.**StoichVariationProcess** (implements sbrt.kernel.MonitoredProcess<R>)
                                                                                                                          - sbrt.kernel.fba.comp.**FbaSolver**- sbrt.shell.io.**FileFormat**- sbrt.shell.text.**FileFormatFormat** (implements sbrt.shell.text.SimpleFormat<T>)- sbrt.shell.io.**FileLineIterator**<E> (implements java.util.Iterator<E>)
                                                                                                                                  - sbrt.shell.io.**SerializedFileIterator**<E>- sbrt.shell.io.**TextFileLineIterator**<E>
                                                                                                                                  - sbrt.kernel.fba.proc.**FluxActivityProcess.Results**- sbrt.kernel.fba.comp.**FluxCapComputer**- sbrt.shell.io.fba.**FluxCapInputFile** (implements sbrt.shell.io.MapTextInputFile<K,V>)- sbrt.kernel.fba.**FluxConstraints**- sbrt.kernel.fba.analysis.**FluxIntervalAnalyzer**- sbrt.shell.io.fba.**FluxIntervalInputFile** (implements sbrt.shell.io.InputFile<E>)- sbrt.kernel.fba.proc.**FluxPlasticityProcess.Results**- sbrt.ext.glpk.**GlpkSolver**<R,C> (implements sbrt.kernel.math.optimization.LinearProgramSolver<R,C>)- sbrt.kernel.math.geom.comp.**Hamming**- sbrt.kernel.math.geom.**HashConvexPolytope**<V> (implements sbrt.kernel.math.geom.ConvexPolytope<V>)- sbrt.kernel.math.geom.**HashConvexPolytopeOld**<V> (implements sbrt.kernel.math.geom.ConvexPolytope<V>)- sbrt.kernel.math.algebra.**HashLinearSystemSolution**<V> (implements sbrt.kernel.math.algebra.LinearSystemSolution<V>)- sbrt.kernel.math.algebra.**IndexPair**<R,C>- sbrt.shell.text.math.**IndexPairFormatV1**<R,C> (implements sbrt.shell.text.math.IndexPairFormat<R,C>)- sbrt.kernel.fba.comp.**InitialPointComputer** (implements sbrt.kernel.fba.comp.FluxCvComputer)- sbrt.kernel.fba.comp.**InitialPointComputer.Results**- sbrt.shell.text.**IntegerFormatV1** (implements sbrt.shell.text.IntegerFormat)- sbrt.kernel.math.**Interval** (implements java.io.Serializable)- sbrt.kernel.math.**IntervalComparer**- sbrt.kernel.math.**IntervalComparer.Diff**<V>- sbrt.kernel.utilities.proc.**IntervalComparison**<V> (implements sbrt.kernel.MonitoredProcess<R>)- sbrt.kernel.math.**IntervalEquiv** (implements sbrt.kernel.math.EquivTester<T>)- sbrt.shell.text.math.**IntervalFormatV1** (implements sbrt.shell.text.math.IntervalFormat)- sbrt.kernel.fba.**IrrevRxnFluxomeV1** (implements sbrt.kernel.fba.IrrevRxnFluxome)- sbrt.shell.text.chem.**IrrevRxnFormatV1** (implements sbrt.shell.text.SimpleFormat<T>)- sbrt.shell.text.fba.**IrrevRxnFormatV1** (implements sbrt.shell.text.SimpleFormat<T>)- sbrt.shell.text.chem.**IrrevRxnFormatV2** (implements sbrt.shell.text.SimpleFormat<T>)- sbrt.kernel.math.stat.comp.**KendallTau**<V> (implements sbrt.kernel.math.stat.comp.Correlator<V,T>)
                                                                                                                                                                                          - sbrt.kernel.math.stat.comp.**KendallTauA**<V>- sbrt.kernel.math.stat.comp.**KendallTauB**<V>
                                                                                                                                                                                          - sbrt.shell.text.math.**LinCombTermFormatV1**<V> (implements sbrt.shell.text.math.LinearCombTermFormat<V>)- sbrt.shell.text.math.**LinCombTermFormatV2**<V> (implements sbrt.shell.text.math.LinearCombTermFormat<V>)- sbrt.shell.text.math.**LinearCombFormatV1**<V> (implements sbrt.shell.text.math.LinearCombFormat<V>)- sbrt.shell.text.math.**LinearCombFormatV2**<V> (implements sbrt.shell.text.math.LinearCombFormat<V>)- sbrt.kernel.math.algebra.proc.**LinearSolverProcess**<V> (implements sbrt.kernel.KernelProcess<T>)- sbrt.shell.text.**LongFormatV1** (implements sbrt.shell.text.LongFormat)- sbrt.kernel.math.stat.comp.**MannWhitneyResults**- sbrt.kernel.math.stat.comp.**MannWhitneyU**- sbrt.shell.mng.**MapManagerInput**<K,V> (implements sbrt.shell.mng.ManagerInput<K,V>)- sbrt.ext.mma.algebra.**MathematicaSolver**<V> (implements sbrt.kernel.math.algebra.LinearSystemSolver<V>)- sbrt.kernel.math.algebra.**MatrixElement**<R,C,E>- sbrt.shell.text.math.**MatrixElementFormatV1**<R,C> (implements sbrt.shell.text.math.MatrixElementFormat<R,C,E>)- sbrt.kernel.math.algebra.**MatrixMultiplier**- sbrt.kernel.math.algebra.**MatrixTransposer**- sbrt.kernel.math.algebra.**MatrixWrap**<R,C,E> (implements sbrt.kernel.math.algebra.Matrix<R,C,E>)
                                                                                                                                                                                                                        - sbrt.kernel.math.algebra.**DoubleMatrixWrap**<R,C> (implements sbrt.kernel.math.algebra.DoubleMatrix<R,C>)
                                                                                                                                                                                                                          - sbrt.kernel.math.algebra.**SparseDoubleMatrixWrap**<R,C> (implements sbrt.kernel.math.algebra.SparseDoubleMatrix<R,C>)
                                                                                                                                                                                                                        - sbrt.shell.proc.fba.**MetatoolFileWriter**- sbrt.ext.mma.algebra.**MMaExprInterpretter**- sbrt.kernel.fba.comp.**MsFluxomeReducer** (implements sbrt.kernel.fba.comp.FluxomeReducer<T>)- sbrt.shell.io.**MultiOutputFiles**<F,R> (implements sbrt.kernel.io.ResultsManager<T>)- sbrt.kernel.math.stat.proc.**MultipleCorrelationAnalysis**<V,T> (implements sbrt.kernel.MonitoredProcess<R>)- sbrt.kernel.math.stat.proc.**MwUAnalysis**<V> (implements sbrt.kernel.KernelProcess<T>)- sbrt.kernel.fba.proc.**NetworkInfoProcess.Results**- sbrt.shell.text.**NonEmptyStringFormat** (implements sbrt.shell.text.SimpleFormat<T>)- sbrt.kernel.math.stat.**NormalDist**- java.io.OutputStream (implements java.io.Closeable, java.io.Flushable)
                                                                                                                                                                                                                                            - java.io.FilterOutputStream
                                                                                                                                                                                                                                              - java.io.PrintStream (implements java.lang.Appendable, java.io.Closeable)
                                                                                                                                                                                                                                                - sbrt.shell.io.**DevNull**
                                                                                                                                                                                                                                              - sbrt.shell.gui.**TextAreaOutputStream**- sbrt.shell.gui.**TextPaneOutputStream**
                                                                                                                                                                                                                                            - sbrt.shell.text.fba.**PalssonRxnNodeParser**- sbrt.shell.proc.fba.**PalssonSbmlReader**- sbrt.shell.proc.fba.**PalssonSbmlTranslater**- sbrt.kernel.math.geom.**ParallelHyperplanes**<V>- sbrt.kernel.math.graph\_theory.**Path**<N>- sbrt.kernel.math.graph\_theory.comp.**PathFinder**<N>- sbrt.kernel.math.graph\_theory.proc.**PathIdProcess**<N> (implements sbrt.kernel.MonitoredProcess<R>)- sbrt.shell.text.**PercentFormatVersion1** (implements sbrt.shell.text.PercentFormat)- sbrt.shell.**ProcessManagerFactory**- sbrt.**ProcessRunner**- sbrt.shell.**ProgramSolverFactory**- sbrt.ext.R.**R**- sbrt.ext.R.stat.**RCorrelator**<V> (implements sbrt.kernel.math.stat.comp.Correlator<V,T>)
                                                                                                                                                                                                                                                                      - sbrt.ext.R.stat.**RKendall**<V>- sbrt.ext.R.stat.**RPearson**<V>- sbrt.ext.R.stat.**RSpearman**<V>
                                                                                                                                                                                                                                                                      - sbrt.ext.R.stat.**RCorrelator.Result** (implements sbrt.kernel.math.stat.comp.CorrResult)- sbrt.shell.proc.graph\_theory.**RedundantCycleRemoval**- sbrt.shell.text.chem.**RevRxnFormatV1** (implements sbrt.shell.text.SimpleFormat<T>)- sbrt.shell.text.chem.**RevRxnFormatV2** (implements sbrt.shell.text.SimpleFormat<T>)- sbrt.shell.io.fba.**RofoofSource** (implements java.util.Iterator<E>)- sbrt.shell.io.**RomiFileSource**<K,V> (implements java.util.Iterator<E>)- sbrt.shell.io.**RomoFileSource**<K,V> (implements java.util.Iterator<E>)- sbrt.shell.io.fba.**RowOrFbaOptOutputFile** (implements sbrt.shell.io.fba.FbaOptOutputFile)- sbrt.shell.text.fba.**RxnNameExprFormatV1** (implements sbrt.shell.text.fba.RxnNameExprFormat<E,S>)- sbrt.shell.text.chem.**RxnNameFormatV1** (implements sbrt.shell.text.chem.RxnNameFormat<T>)- sbrt.shell.text.fba.**RxnNameListFormatV1** (implements sbrt.shell.text.fba.RxnNameListFormat)- sbrt.shell.text.fba.**RxnNameOrExprFormatV1** (implements sbrt.shell.text.fba.RxnNameOrExprFormat)- sbrt.shell.text.fba.**RxnNameSetFormatV1** (implements sbrt.shell.text.fba.RxnNameSetFormat)- sbrt.shell.text.fba.**RxnNameVerifier** (implements sbrt.shell.text.chem.RxnNameFormat<T>)- sbrt.shell.proc.fba.**SbmlWriter**- sbrt.**Sbrt**- sbrt.shell.text.math.**SenseFormat** (implements sbrt.shell.text.SimpleFormat<T>)- sbrt.shell.io.fba.**SerFbaOptOutputFile** (implements sbrt.shell.io.fba.FbaOptOutputFile)- sbrt.shell.io.**SerializedInputFile**<E> (implements sbrt.shell.io.InputFile<E>)
                                                                                                                                                                                                                                                                                                            - sbrt.shell.io.**SerializedMapInputFile**<K,V> (implements sbrt.shell.io.MapInputFile<K,V>)
                                                                                                                                                                                                                                                                                                            - sbrt.shell.io.**SerializedOutputFile**<E> (implements sbrt.shell.io.OutputFile<T>)
                                                                                                                                                                                                                                                                                                              - sbrt.shell.io.**SmoFile**<K,V> (implements sbrt.shell.io.MapOutputFile<K,V>)
                                                                                                                                                                                                                                                                                                              - sbrt.shell.io.fba.**SfoofSource** (implements java.util.Iterator<E>)- sbrt.shell.text.**SimpleListFormat**<E> (implements sbrt.shell.text.SimpleFormat<T>)- sbrt.shell.proc.fba.**SimpleRxnFileTranslator**- sbrt.shell.text.**SimpleSetFormat**<E> (implements sbrt.shell.text.SimpleFormat<T>)- sbrt.kernel.math.comb.proc.**SingleElementUnionProc**<E> (implements sbrt.kernel.KernelProcess<T>)- sbrt.kernel.math.comb.comp.**SingleElementUnionsV1**- sbrt.kernel.math.comb.comp.**SingleElementUnionsV2**- sbrt.kernel.fba.comp.**SlpCycleComputer** (implements sbrt.kernel.fba.comp.CycleComputer)- sbrt.kernel.fba.comp.**SlpExCurrComputerV2** (implements sbrt.kernel.fba.comp.ExtremeCurrentComputer)- sbrt.shell.io.**SmoFileSource**<K,V> (implements java.util.Iterator<E>)- sbrt.shell.text.**SpecialChars**- sbrt.kernel.math.comb.proc.**StrictSingleElementUnionProc**<E> (implements sbrt.kernel.KernelProcess<T>)- sbrt.kernel.math.comb.comp.**StrictSingleElementUnions**- sbrt.shell.text.**StringFormat** (implements sbrt.shell.text.SimpleFormat<T>)- sbrt.kernel.math.stat.comp.**TauResult** (implements sbrt.kernel.math.stat.comp.CorrResult)- sbrt.kernel.math.geom.comp.**Taxicab**- sbrt.shell.io.**TextInputFile**<E> (implements sbrt.shell.io.InputFile<E>)
                                                                                                                                                                                                                                                                                                                                                - sbrt.shell.io.fba.**RxnNamExprSetInputFile**<E,S>
                                                                                                                                                                                                                                                                                                                                                - sbrt.shell.io.**TextOutputFile**<T> (implements sbrt.shell.io.OutputFile<T>)- java.lang.Throwable (implements java.io.Serializable)
                                                                                                                                                                                                                                                                                                                                                    - java.lang.Exception
                                                                                                                                                                                                                                                                                                                                                      - java.lang.RuntimeException
                                                                                                                                                                                                                                                                                                                                                        - sbrt.shell.**ApplicationException**
                                                                                                                                                                                                                                                                                                                                                          - sbrt.shell.text.**FormatException**
                                                                                                                                                                                                                                                                                                                                                          - sbrt.kernel.io.**ConflictingInputException**- sbrt.kernel.math.**ConstraintViolationException**- sbrt.kernel.math.geom.**DimensionException**- sbrt.kernel.math.**ExceededLimitException**- sbrt.ext.**ExternalSoftwareException**
                                                                                                                                                                                                                                                                                                                                                                    - sbrt.kernel.math.optimization.**ProgramSolverException**
                                                                                                                                                                                                                                                                                                                                                                    - sbrt.**FatalException**- sbrt.kernel.fba.comp.**FbaOptException**- sbrt.kernel.fba.comp.**FluxCapException**- sbrt.kernel.fba.comp.**FluxRangeException**- sbrt.kernel.math.**IllegalBoundsException**- sbrt.kernel.math.algebra.**IllegalFreeVariableException**- sbrt.kernel.math.**InfeasibleSolutionException**- sbrt.kernel.fba.comp.**InvalidFluxVectorException**- sbrt.kernel.math.**InvalidPointException**- sbrt.ext.R.**JriException**- sbrt.ext.mma.**MathematicaException**- sbrt.kernel.math.**MissingVariableException**- sbrt.kernel.math.**UnboundedVariableException**
                                                                                                                                                                                                                                                                                                                                                    - sbrt.shell.text.**TrueFalseFormat** (implements sbrt.shell.text.BooleanFormat)- sbrt.kernel.util.**UnmodifiableCollection**<E> (implements java.util.Collection<E>)- sbrt.kernel.util.**UnmodifiableIterator**<E> (implements java.util.Iterator<E>)- sbrt.kernel.util.**UnmodifiableList**<E> (implements java.util.List<E>)- sbrt.kernel.util.**UnmodifiableMap**<K,V> (implements java.util.Map<K,V>)- sbrt.kernel.util.**UnmodifiableSet**<E> (implements java.util.Set<E>)- sbrt.kernel.util.**Util**- sbrt.kernel.utilities.proc.**VariableParticipation**<E,V> (implements sbrt.kernel.KernelProcess<T>)- sbrt.kernel.math.**VectorComparer**- sbrt.kernel.math.**VectorComparer.Diff**<V>- sbrt.kernel.utilities.proc.**VectorComparison**<V> (implements sbrt.kernel.MonitoredProcess<R>)- sbrt.kernel.math.algebra.proc.**VectorIntersection**<K,V> (implements sbrt.kernel.KernelProcess<T>)- sbrt.kernel.io.**WarningHandlerFactory**- sbrt.shell.io.**WorkingDirectory**- sbrt.kernel.fba.comp.**WwFluxomeReducer** (implements sbrt.kernel.fba.comp.FluxomeReducer<T>)- sbrt.shell.io.**XmlFileFactory**- sbrt.shell.text.**YesNoFormat** (implements sbrt.shell.text.BooleanFormat)

## Interface Hierarchy

- sbrt.kernel.fba.**ChemRxn**- sbrt.kernel.math.geom.**ConvexPolytope**<V>- sbrt.kernel.math.stat.comp.**Correlator**<V,T>- sbrt.kernel.math.stat.comp.**CorrResult**- sbrt.kernel.fba.comp.**CycleComputer**- sbrt.kernel.math.**EquivTester**<T>- sbrt.kernel.fba.comp.**ExtremeCurrentComputer**- sbrt.kernel.fba.comp.**FluxCvComputer**
                - sbrt.kernel.fba.comp.**FluxIntervalComputer**
                - sbrt.kernel.fba.**Fluxome**
                  - sbrt.kernel.fba.**CatalyzedFluxome**
                    - sbrt.kernel.fba.**IrrevRxnFluxome**
                    - sbrt.kernel.fba.**IrrevRxnFluxome**
                  - sbrt.kernel.fba.comp.**FluxomeReducer**<T>- sbrt.shell.text.**Format**
                      - sbrt.shell.text.**AppInputFileLineFormat**- sbrt.shell.text.**BooleanFormat**- sbrt.shell.text.chem.**CatalystFormat**- sbrt.shell.text.fba.**CatalystListFormat**- sbrt.shell.text.fba.**CatalystSetFormat**- sbrt.shell.text.chem.**ChemSpeciesFormat**- sbrt.shell.text.math.**CoeffFormat**- sbrt.shell.text.**CollectionFormat**<E>- sbrt.shell.text.fba.**ConstraintsFileLineFormat**<E>- sbrt.shell.text.math.**DirEdgeFormat**<N>- sbrt.shell.text.**DoubleFormat**- sbrt.shell.text.fba.**FbaOptFileLineFormat**- sbrt.shell.text.fba.**FluxCapFormat**- sbrt.shell.text.fba.**FluxomeSolutionFileLineFormat**- sbrt.shell.text.fba.**FluxVectorFormat**- sbrt.shell.text.math.**IndexPairFormat**<R,C>- sbrt.shell.text.**IntegerFormat**- sbrt.shell.text.math.**IntervalFormat**- sbrt.shell.text.math.**LinearCombFormat**<V>- sbrt.shell.text.math.**LinearCombTermFormat**<V>- sbrt.shell.text.**LongFormat**- sbrt.shell.text.**MapEntryFormat**<K,V>- sbrt.shell.text.**MapFormat**<K,V>
                                                                    - sbrt.shell.text.**AppInputFileLineFormat**- sbrt.shell.text.fba.**ConstraintsFileLineFormat**<E>- sbrt.shell.text.fba.**FbaOptFileLineFormat**- sbrt.shell.text.fba.**FluxCapFormat**- sbrt.shell.text.fba.**FluxomeSolutionFileLineFormat**- sbrt.shell.text.fba.**FluxVectorFormat**
                                                                    - sbrt.shell.text.math.**MathExprFormat**<E,V>
                                                                      - sbrt.shell.text.math.**LinearCombFormat**<V>
                                                                      - sbrt.shell.text.math.**MatrixElementFormat**<R,C,E>- sbrt.shell.text.**PercentFormat**- sbrt.shell.text.fba.**RxnNameExprFormat**<E,S>- sbrt.shell.text.chem.**RxnNameFormat**<T>- sbrt.shell.text.fba.**RxnNameListFormat**- sbrt.shell.text.fba.**RxnNameOrExprFormat**- sbrt.shell.text.fba.**RxnNameSetFormat**- sbrt.shell.text.**SimpleFormat**<T> (also extends sbrt.shell.text.Formatter<T>, sbrt.shell.text.Parser<T>)
                                                                                      - sbrt.shell.text.**BooleanFormat**- sbrt.shell.text.chem.**CatalystFormat**- sbrt.shell.text.fba.**CatalystListFormat**- sbrt.shell.text.fba.**CatalystSetFormat**- sbrt.shell.text.chem.**ChemSpeciesFormat**- sbrt.shell.text.math.**CoeffFormat**- sbrt.shell.text.**CollectionFormat**<E>- sbrt.shell.text.math.**DirEdgeFormat**<N>- sbrt.shell.text.**DoubleFormat**- sbrt.shell.text.math.**IndexPairFormat**<R,C>- sbrt.shell.text.**IntegerFormat**- sbrt.shell.text.math.**IntervalFormat**- sbrt.shell.text.math.**LinearCombFormat**<V>- sbrt.shell.text.math.**LinearCombTermFormat**<V>- sbrt.shell.text.**LongFormat**- sbrt.shell.text.math.**MathExprFormat**<E,V>
                                                                                                                      - sbrt.shell.text.math.**LinearCombFormat**<V>
                                                                                                                      - sbrt.shell.text.math.**MatrixElementFormat**<R,C,E>- sbrt.shell.text.**PercentFormat**- sbrt.shell.text.fba.**RxnNameExprFormat**<E,S>- sbrt.shell.text.chem.**RxnNameFormat**<T>- sbrt.shell.text.fba.**RxnNameListFormat**- sbrt.shell.text.fba.**RxnNameOrExprFormat**- sbrt.shell.text.fba.**RxnNameSetFormat**
                      - sbrt.shell.text.**Formatter**<T>
                        - sbrt.shell.text.**BooleanFormat**- sbrt.shell.text.chem.**CatalystFormat**- sbrt.shell.text.fba.**CatalystListFormat**- sbrt.shell.text.fba.**CatalystSetFormat**- sbrt.shell.text.chem.**ChemSpeciesFormat**- sbrt.shell.text.math.**CoeffFormat**- sbrt.shell.text.**CollectionFormat**<E>- sbrt.shell.text.math.**CorrResultsFormatter**<T>- sbrt.shell.text.math.**DirEdgeFormat**<N>- sbrt.shell.text.**DoubleFormat**- sbrt.shell.text.fba.**FbaOptHeaderFormatter**- sbrt.shell.text.math.**IndexPairFormat**<R,C>- sbrt.shell.text.**IntegerFormat**- sbrt.shell.text.math.**IntervalFormat**- sbrt.shell.text.math.**LinearCombFormat**<V>- sbrt.shell.text.math.**LinearCombTermFormat**<V>- sbrt.shell.text.**LinkedHashSetFormat**<E> (also extends sbrt.shell.text.Parser<T>)- sbrt.shell.text.**ListFormat**<E> (also extends sbrt.shell.text.Parser<T>)- sbrt.shell.text.**LongFormat**- sbrt.shell.text.math.**MathExprFormat**<E,V>
                                                                - sbrt.shell.text.math.**LinearCombFormat**<V>
                                                                - sbrt.shell.text.math.**MatrixElementFormat**<R,C,E>- sbrt.shell.text.**PercentFormat**- sbrt.shell.text.fba.**RxnNameExprFormat**<E,S>- sbrt.shell.text.chem.**RxnNameFormat**<T>- sbrt.shell.text.fba.**RxnNameListFormat**- sbrt.shell.text.fba.**RxnNameOrExprFormat**- sbrt.shell.text.fba.**RxnNameSetFormat**- sbrt.shell.text.**SimpleFormat**<T> (also extends sbrt.shell.text.Format, sbrt.shell.text.Parser<T>)
                                                                                - sbrt.shell.text.**BooleanFormat**- sbrt.shell.text.chem.**CatalystFormat**- sbrt.shell.text.fba.**CatalystListFormat**- sbrt.shell.text.fba.**CatalystSetFormat**- sbrt.shell.text.chem.**ChemSpeciesFormat**- sbrt.shell.text.math.**CoeffFormat**- sbrt.shell.text.**CollectionFormat**<E>- sbrt.shell.text.math.**DirEdgeFormat**<N>- sbrt.shell.text.**DoubleFormat**- sbrt.shell.text.math.**IndexPairFormat**<R,C>- sbrt.shell.text.**IntegerFormat**- sbrt.shell.text.math.**IntervalFormat**- sbrt.shell.text.math.**LinearCombFormat**<V>- sbrt.shell.text.math.**LinearCombTermFormat**<V>- sbrt.shell.text.**LongFormat**- sbrt.shell.text.math.**MathExprFormat**<E,V>
                                                                                                                - sbrt.shell.text.math.**LinearCombFormat**<V>
                                                                                                                - sbrt.shell.text.math.**MatrixElementFormat**<R,C,E>- sbrt.shell.text.**PercentFormat**- sbrt.shell.text.fba.**RxnNameExprFormat**<E,S>- sbrt.shell.text.chem.**RxnNameFormat**<T>- sbrt.shell.text.fba.**RxnNameListFormat**- sbrt.shell.text.fba.**RxnNameOrExprFormat**- sbrt.shell.text.fba.**RxnNameSetFormat**
                        - java.lang.Iterable<T>
                          - sbrt.shell.io.**InputFile**<E> (also extends sbrt.shell.io.SbrtFile)
                            - sbrt.shell.io.**MapInputFile**<K,V>
                              - sbrt.shell.io.**MapTextInputFile**<K,V> (also extends sbrt.shell.io.MapTextFile<K,V>)
                              - sbrt.shell.io.**MapTextInputFile**<K,V> (also extends sbrt.shell.io.MapInputFile<K,V>, sbrt.shell.io.MapTextFile<K,V>)
                            - sbrt.kernel.math.**LinearComb**<V> (also extends sbrt.kernel.math.Polynomial<V>)- sbrt.kernel.math.**LinearComb.Term**<V>- sbrt.shell.io.**MapInputFile**<K,V>
                                  - sbrt.shell.io.**MapTextInputFile**<K,V> (also extends sbrt.shell.io.MapTextFile<K,V>)
                                  - sbrt.shell.io.**MapTextInputFile**<K,V> (also extends sbrt.shell.io.MapInputFile<K,V>, sbrt.shell.io.MapTextFile<K,V>)- sbrt.kernel.math.**Polynomial.Term**<V>
                                      - sbrt.kernel.math.**LinearComb.Term**<V>- sbrt.kernel.math.**QuadraticExpr.Term**<V>
                                      - sbrt.kernel.math.**QuadraticExpr.Term**<V>
                          - sbrt.kernel.**KernelProcess**<T>
                            - sbrt.kernel.**MonitoredProcess**<R>
                            - sbrt.kernel.math.algebra.**LinearSystemSolution**<V>- sbrt.kernel.math.algebra.**LinearSystemSolver**<V>- sbrt.shell.mng.**ManagerInput**<K,V>- sbrt.kernel.math.**MathExpr**<V>
                                    - sbrt.kernel.math.**LinearComb**<V> (also extends java.lang.Iterable<T>, sbrt.kernel.math.Polynomial<V>)- sbrt.kernel.math.**Polynomial**<V>
                                        - sbrt.kernel.math.**LinearComb**<V> (also extends java.lang.Iterable<T>)- sbrt.kernel.math.**QuadraticExpr**<V>
                                        - sbrt.kernel.math.**QuadraticExpr**<V>
                                    - sbrt.kernel.math.algebra.**Matrix**<R,C,E>
                                      - sbrt.kernel.math.algebra.**DoubleMatrix**<R,C>
                                        - sbrt.kernel.math.algebra.**DoubleMatrixBuilder**<R,C> (also extends sbrt.kernel.math.algebra.MatrixBuilder<R,C,E>)
                                          - sbrt.kernel.math.algebra.**SmdMatrix**<R,C> (also extends sbrt.kernel.math.algebra.SparseDoubleMatrix<R,C>)
                                          - sbrt.kernel.math.algebra.**SmdMatrix**<R,C> (also extends sbrt.kernel.math.algebra.DoubleMatrixBuilder<R,C>, sbrt.kernel.math.algebra.SparseDoubleMatrix<R,C>)- sbrt.kernel.math.algebra.**SparseDoubleMatrix**<R,C>
                                              - sbrt.kernel.math.algebra.**SmdMatrix**<R,C> (also extends sbrt.kernel.math.algebra.DoubleMatrixBuilder<R,C>)
                                        - sbrt.kernel.math.algebra.**DoubleMatrixBuilder**<R,C> (also extends sbrt.kernel.math.algebra.DoubleMatrix<R,C>, sbrt.kernel.math.algebra.MatrixBuilder<R,C,E>)
                                          - sbrt.kernel.math.algebra.**SmdMatrix**<R,C> (also extends sbrt.kernel.math.algebra.SparseDoubleMatrix<R,C>)
                                          - sbrt.kernel.math.algebra.**MatrixBuilder**<R,C,E>
                                            - sbrt.kernel.math.algebra.**DoubleMatrixBuilder**<R,C> (also extends sbrt.kernel.math.algebra.DoubleMatrix<R,C>)
                                              - sbrt.kernel.math.algebra.**SmdMatrix**<R,C> (also extends sbrt.kernel.math.algebra.SparseDoubleMatrix<R,C>)
                                              - sbrt.kernel.math.algebra.**SmdMatrix**<R,C> (also extends sbrt.kernel.math.algebra.DoubleMatrixBuilder<R,C>, sbrt.kernel.math.algebra.SparseDoubleMatrix<R,C>)
                                            - sbrt.kernel.math.algebra.**SmdMatrix**<R,C> (also extends sbrt.kernel.math.algebra.DoubleMatrixBuilder<R,C>, sbrt.kernel.math.algebra.SparseDoubleMatrix<R,C>)- sbrt.kernel.math.algebra.**SparseDoubleMatrix**<R,C>
                                                - sbrt.kernel.math.algebra.**SmdMatrix**<R,C> (also extends sbrt.kernel.math.algebra.DoubleMatrixBuilder<R,C>)
                                      - sbrt.shell.text.**Parser**<T>
                                        - sbrt.shell.text.**BooleanFormat**- sbrt.shell.text.chem.**CatalystFormat**- sbrt.shell.text.fba.**CatalystListFormat**- sbrt.shell.text.fba.**CatalystSetFormat**- sbrt.shell.text.chem.**ChemSpeciesFormat**- sbrt.shell.text.math.**CoeffFormat**- sbrt.shell.text.**CollectionFormat**<E>- sbrt.shell.text.math.**DirEdgeFormat**<N>- sbrt.shell.text.**DoubleFormat**- sbrt.shell.text.fba.**FbaOptHeaderParser**- sbrt.shell.text.math.**IndexPairFormat**<R,C>- sbrt.shell.text.**IntegerFormat**- sbrt.shell.text.math.**IntervalFormat**- sbrt.shell.text.math.**LinearCombFormat**<V>- sbrt.shell.text.math.**LinearCombTermFormat**<V>- sbrt.shell.text.**LinkedHashSetFormat**<E> (also extends sbrt.shell.text.Formatter<T>)- sbrt.shell.text.**ListFormat**<E> (also extends sbrt.shell.text.Formatter<T>)- sbrt.shell.text.**LongFormat**- sbrt.shell.text.math.**MathExprFormat**<E,V>
                                                                              - sbrt.shell.text.math.**LinearCombFormat**<V>
                                                                              - sbrt.shell.text.math.**MatrixElementFormat**<R,C,E>- sbrt.shell.text.**PercentFormat**- sbrt.shell.text.fba.**RxnNameExprFormat**<E,S>- sbrt.shell.text.chem.**RxnNameFormat**<T>- sbrt.shell.text.fba.**RxnNameListFormat**- sbrt.shell.text.fba.**RxnNameOrExprFormat**- sbrt.shell.text.fba.**RxnNameSetFormat**- sbrt.shell.text.**SimpleFormat**<T> (also extends sbrt.shell.text.Format, sbrt.shell.text.Formatter<T>)
                                                                                              - sbrt.shell.text.**BooleanFormat**- sbrt.shell.text.chem.**CatalystFormat**- sbrt.shell.text.fba.**CatalystListFormat**- sbrt.shell.text.fba.**CatalystSetFormat**- sbrt.shell.text.chem.**ChemSpeciesFormat**- sbrt.shell.text.math.**CoeffFormat**- sbrt.shell.text.**CollectionFormat**<E>- sbrt.shell.text.math.**DirEdgeFormat**<N>- sbrt.shell.text.**DoubleFormat**- sbrt.shell.text.math.**IndexPairFormat**<R,C>- sbrt.shell.text.**IntegerFormat**- sbrt.shell.text.math.**IntervalFormat**- sbrt.shell.text.math.**LinearCombFormat**<V>- sbrt.shell.text.math.**LinearCombTermFormat**<V>- sbrt.shell.text.**LongFormat**- sbrt.shell.text.math.**MathExprFormat**<E,V>
                                                                                                                              - sbrt.shell.text.math.**LinearCombFormat**<V>
                                                                                                                              - sbrt.shell.text.math.**MatrixElementFormat**<R,C,E>- sbrt.shell.text.**PercentFormat**- sbrt.shell.text.fba.**RxnNameExprFormat**<E,S>- sbrt.shell.text.chem.**RxnNameFormat**<T>- sbrt.shell.text.fba.**RxnNameListFormat**- sbrt.shell.text.fba.**RxnNameOrExprFormat**- sbrt.shell.text.fba.**RxnNameSetFormat**
                                        - sbrt.shell.mng.**ProcessManager**<K,V>
                                          - sbrt.shell.mng.**KernelProcessManager**<K,V>- sbrt.shell.mng.**ShellProcessManager**<K,V>
                                          - sbrt.kernel.math.optimization.**ProgramSolver**<R,C>
                                            - sbrt.kernel.math.optimization.**LinearProgramSolver**<R,C>- sbrt.kernel.math.optimization.**QuadraticProgramSolver**<R,C>
                                            - sbrt.kernel.util.**ProgressMonitor**
                                              - sbrt.kernel.util.**MutableProgressMonitor**
                                              - sbrt.kernel.io.**ResultsManager**<T>
                                                - sbrt.shell.io.fba.**FbaOptOutputFile**- sbrt.shell.io.**MapOutputFile**<K,V>
                                                    - sbrt.shell.io.**MapTextOutputFile**<K,V> (also extends sbrt.shell.io.MapTextFile<K,V>)
                                                    - sbrt.shell.io.**MapTextOutputFile**<K,V> (also extends sbrt.shell.io.MapOutputFile<K,V>, sbrt.shell.io.MapTextFile<K,V>)- sbrt.shell.io.**OutputFile**<T> (also extends sbrt.shell.io.SbrtFile)
                                                        - sbrt.shell.io.fba.**FbaOptOutputFile**- sbrt.shell.io.**MapOutputFile**<K,V>
                                                            - sbrt.shell.io.**MapTextOutputFile**<K,V> (also extends sbrt.shell.io.MapTextFile<K,V>)
                                                            - sbrt.shell.io.**MapTextOutputFile**<K,V> (also extends sbrt.shell.io.MapOutputFile<K,V>, sbrt.shell.io.MapTextFile<K,V>)
                                                - sbrt.shell.io.**SbrtFile**
                                                  - sbrt.shell.io.fba.**FbaOptOutputFile**- sbrt.shell.io.**InputFile**<E> (also extends java.lang.Iterable<T>)
                                                      - sbrt.shell.io.**MapInputFile**<K,V>
                                                        - sbrt.shell.io.**MapTextInputFile**<K,V> (also extends sbrt.shell.io.MapTextFile<K,V>)
                                                        - sbrt.shell.io.**MapTextInputFile**<K,V> (also extends sbrt.shell.io.MapInputFile<K,V>, sbrt.shell.io.MapTextFile<K,V>)
                                                      - sbrt.shell.io.**MapInputFile**<K,V>
                                                        - sbrt.shell.io.**MapTextInputFile**<K,V> (also extends sbrt.shell.io.MapTextFile<K,V>)
                                                        - sbrt.shell.io.**MapOutputFile**<K,V>
                                                          - sbrt.shell.io.**MapTextOutputFile**<K,V> (also extends sbrt.shell.io.MapTextFile<K,V>)
                                                          - sbrt.shell.io.**MapTextFile**<K,V>
                                                            - sbrt.shell.io.**MapTextInputFile**<K,V> (also extends sbrt.shell.io.MapInputFile<K,V>)- sbrt.shell.io.**MapTextOutputFile**<K,V> (also extends sbrt.shell.io.MapOutputFile<K,V>)
                                                            - sbrt.shell.io.**MapTextInputFile**<K,V> (also extends sbrt.shell.io.MapInputFile<K,V>, sbrt.shell.io.MapTextFile<K,V>)- sbrt.shell.io.**MapTextOutputFile**<K,V> (also extends sbrt.shell.io.MapOutputFile<K,V>, sbrt.shell.io.MapTextFile<K,V>)- sbrt.shell.io.**OutputFile**<T> (also extends sbrt.kernel.io.ResultsManager<T>)
                                                                  - sbrt.shell.io.fba.**FbaOptOutputFile**- sbrt.shell.io.**MapOutputFile**<K,V>
                                                                      - sbrt.shell.io.**MapTextOutputFile**<K,V> (also extends sbrt.shell.io.MapTextFile<K,V>)
                                                                      - sbrt.shell.io.**MapTextOutputFile**<K,V> (also extends sbrt.shell.io.MapOutputFile<K,V>, sbrt.shell.io.MapTextFile<K,V>)
                                                  - sbrt.kernel.io.**WarningHandler**

## Enum Hierarchy

- java.lang.Object
  - java.lang.Enum<E> (implements java.lang.Comparable<T>, java.io.Serializable)
    - sbrt.kernel.math.optimization.**OptimizationSense**- sbrt.kernel.math.optimization.**ProgramSolverStatus**

---


|  |  |  |
| --- | --- | --- |
| **Overview** \| Package \| Class \| Use \| **Tree** \| **Deprecated** \| **Index** \| **Help** | |  |
| PREV   NEXT | **FRAMES**    **NO FRAMES**     **All Classes** |  |


---
